# Isolated Aortic Valve Replacement: The Impact of Patient-Prosthesis Mismatch on Early Mortality

**DOI:** 10.7759/cureus.82514

**Published:** 2025-04-18

**Authors:** Selman Dumani, Alessia Mehmeti, Ermal Likaj, Laureta Dibra, Alfred Ibrahimi, Edlira Rruci, Stavri Llazo, Devis Pellumbi, Vera Beca, Ali Refatllari, Elizana Zaimi (Petrela), Altin Veshti

**Affiliations:** 1 Cardiac Surgery, University Hospital Center "Mother Teresa", Tirana, ALB; 2 Anesthesiology, University Hospital Center “Mother Teresa”, Tirana, ALB; 3 Nosocomial Infections, University Hospital Obstetrics and Gynecology "Queen Geraldina", Tirana, ALB; 4 Public Health, Faculty of Medicine, University of Medicine, Tirana, ALB

**Keywords:** aortic valve, aortic valve replacement, aortic valve surgery, mortality, patient-prosthesis mismatch

## Abstract

Patient-prosthesis mismatch (PPM) occurs when the effective orifice area (EOA) of a prosthetic heart valve is too small relative to the patient’s body size, leading to elevated postoperative gradients and potentially adverse clinical outcomes. It remains a significant topic of concern despite advances in prosthesis manufacturing technologies.

The primary objective of this study was to determine the prevalence of PPM and assess its impact on early (in-hospital) mortality following isolated surgical aortic valve replacement (AVR).

This retrospective study included 491 adult patients (≥18 years) who underwent isolated surgical AVR at University Hospital Center “Mother Teresa” in Tirana, Albania, from January 2007 to December 2023. Patients undergoing concomitant procedures (e.g., coronary artery bypass grafting (CABG), mitral surgery) were excluded. Both mechanical and bioprosthetic valves were included. Data were collected on general demographic characteristics, important intraoperative and postoperative times, and postoperative outcomes. Early mortality was defined as any in-hospital death occurring after the intervention. The indexed EOA (EOA-i) was used to classify PPM as severe (EOA-i < 0.65 cm²/m²), moderate (0.65 ˂ EOA-i  ≤ 0.85 cm²/m²), or none (EOA-i > 0.85 cm²/m²). EOA-i was calculated using prosthesis-specific reference EOAs provided by valve manufacturers. Mortality were assessed in relation to PPM severity.

Our study included 491 patients with a mean age of 62.28 ± 10.76 years. The majority of patients group (63.3%) were male, and 91.8% of the procedures were elective. Among them, 44.4% had moderate PPM and 11.0% had severe PPM. A total of eight early deaths (1.6%) occurred. Early mortality was significantly higher in the severe PPM group (3.7%) compared to the moderate (1.8%) and no PPM groups (0.9%) (p = 0.048, Fisher’s exact test). In multivariate logistic regression, severe PPM was associated with increased odds of early mortality (odds ratio (OR) 15.62, 95% confidence interval (CI) 9.004-21.10, p = 0.050) after adjusting for valve type, body size, age and New York Heart Association (NYHA) class.

Severe PPM is strongly associated with increased short-term mortality following AVR. Implementing strategies to prevent PPM such as CT-based annulus sizing and annular enlargement during surgery is crucial for reducing postoperative mortality risks.

## Introduction

Surgical aortic valve (AV) replacement (AVR) is the gold standard treatment for patients with severe AV disease, such as aortic stenosis or regurgitation, and represents the most frequently performed heart valve surgery in cardiac surgery centers. In our country, the number of patients requiring surgery for AV disease continues to rise as a result of increased life expectancy. While the benefits of AVR in terms of symptom relief and survival improvement are well-established, the procedure is sometimes complicated by patient-prosthesis mismatch (PPM), a concept first introduced by Rahimtoola et al. in 1978 [1]. This is a condition in which the effective area of the prosthetic valve, after insertion into the patient, is smaller than that of a normal human valve [1]. On the other hand, this problem can be prevented at the time of prosthesis insertion.

Despite advancements in surgical techniques and prosthetic valve design, PPM remains a significant concern in AVR. PPM results in persistently elevated transvalvular gradients, potentially leading to residual pressure overload, impaired left ventricular regression, and adverse early or late clinical outcomes [2]. Epidemiological data indicate that PPM is a common occurrence among patients undergoing AVR, with large meta-analyses reporting a PPM incidence of over 50% [3,4].

The clinical implications of PPM are profound, with research demonstrating its adverse effects on both short-term and long-term outcomes [5-7]. While studies have established the prevalence and implications of PPM, its impact on early (in-hospital) mortality remains controversial, especially in populations with limited local data, such as ours. Therefore, this study aimed to determine the prevalence of PPM in patients undergoing isolated AVR at our center and assess the impact of this phenomenon on short-term mortality.

## Materials and methods

Study design

This retrospective study included 491 adult patients (≥18 years) who underwent isolated surgical AVR at the University Hospital Center “Mother Teresa,” a tertiary referral center with a high annual volume of cardiac surgeries. Patients undergoing combined cardiac procedures (e.g., AVR with coronary artery bypass grafting (CABG) or mitral valve surgery), patients who had reoperative AVR (redo procedures), and patients with incomplete or missing postoperative outcome data in medical records were excluded from this study. Standard surgical techniques were employed. Both mechanical and bioprosthetic valves were implanted based on patient characteristics, age, and clinical indications

The objective was to evaluate early postoperative outcomes and the impact of PPM in adult patients undergoing isolated AVR. The study period spanned from January 1, 2007, to December 31, 2023. The study protocol was reviewed and approved by the institutional ethics committee. Due to the retrospective nature of the study, the requirement for informed consent was waived. Patient data were retrospectively extracted from the hospital’s medical record database. The collected variables included demographic information, such as age, sex, and body surface area (BSA); operative details including the type and size of the implanted prosthetic valve; and the indexed effective orifice area (EOA-i), which was calculated by indexing the effective orifice area (EOA) of each valve, sourced from manufacturer reference tables, to the patient's BSA [8]. PPM was classified as none (> 0.85 cm²/m²), moderate (0.65-0.85 cm²/m²), or severe (< 0.65 cm²/m²). The primary early postoperative outcome assessed was in-hospital mortality, defined as death occurring before hospital discharge, irrespective of the time frame. The relationship between PPM and early postoperative mortality was the central focus of the analysis.

Statistical analysis

All statistical analyses were conducted using IBM SPSS Statistics for Windows, version 21 (IBM Corp., Armonk, USA).

Descriptive statistics were used to summarize patient characteristics and outcomes, expressed as means with SDs for continuous variables and percentages for categorical variables.

Comparative analysis between PPM groups (no, moderate, and severe) was performed to assess differences in early outcomes. Between-group comparisons used Fisher’s exact test.

Multivariate logistic regression analysis was employed to identify independent predictors of early mortality and included covariates with clinical relevance or statistical significance in univariate analysis: severe PPM, moderate PPM, left ventricle ejection fraction (LVEF), cross-clamping time and gender. Adjustments were made for valve type, New York Heart Association (NYHA) class, body size and age. Model calibration was assessed using the Hosmer-Lemeshow test, and model discrimination via c-statistic. Missing data were handled with listwise deletion.

Results were reported as odds ratios (ORs) with 95% confidence intervals (CIs) and associated p-values, with statistical significance set at p < 0.05.

## Results

A total of 491 patients treated with isolated AVR were included in the study.. They had a mean (SD) age of 62.28 (10.76) years and were predominantly male (63.3%). Table 1 presents the collected demographic data. The majority of patients (91.2%) underwent elective surgery, while 8.8% required urgent interventions. At hospital admission, most of the patients were classified as NYHA Class III (54.2%) or Class II (39.90%), reflecting moderate to severe heart failure symptoms. Only a small proportion were categorized as Class I (2.65%) or Class IV (4.25%). The most common AV pathology was aortic stenosis (72.5%), followed by aortic regurgitation (14.6%) and mixed pathology (12.9%). The most prevalent comorbidity was arterial hypertension (71.6%), followed by diabetes mellitus (17.4%).

**Table 1:** General data Mixed Pathology includes AV regurgitation and AV stenosis. NYHA: New York Heart Association; COPD: Chronic obstructive pulmonary disease; PsAP: Pulmonary systolic artery pressure; EF: Ejection fraction; AV: Aortic valve

Data	Number of Patients (n) and (%)	Percentage (%); Mean ± SD
Age (years)	491 (100%)	62.28 ± 10.76
Male	311 (63.34 %)	63.34%
Female	180	36.66%
Elective	447	91.0%
Urgent	44	9.0%
NYHA Class I	13	2.65%
NYHA Class II	191	38.9%
NYHA Class III	266	54.20%
NYHA Class IV	21	4.25%
AV Stenosis	356	72.5%
AV Regurgitation	72	14.6%
Mixed Pathology	63	12.9%
Arterial Hypertension	350	71.6%
Diabetes Mellitus	85	17.4%
COPD	18	3.7%
EF (%)	491	60.1±9.18
Annulus Size	463 (94.2%)	21.62±1.98
PsAP	480 (97.7 %)	40.2±15.60

The overall mortality was 1.6%, with the highest rate observed in the patients with severe PPM (3.7%).

Table 2 provides key intraoperative and postoperative data. The average duration of cardiopulmonary bypass (CPB) was 83.85 ± 22.63 minutes, and the mean aortic cross-clamp (XC) time was 65.22 ± 19.20 minutes. Postoperative outcomes included an average intensive care unit stay of 59.59 ± 65.60 hours, mean totally directed respiratory assistance time of 16.12 ± 42.16 hours, and average postoperative hospital stay of 9.15 ± 4.45 days.

**Table 2:** Intraoperative and postoperative times Min: Minutes; CPB: Cardiopulmonary bypass; XC: Aortic cross-clamp

Data	Mean ± SD
CPB (min)	83.85 ± 22.63
XC (min)	65.22 ± 19.20
ICU Stay (hours)	59.59 ± 65.60
Postoperative Hospital Stay (days)	9.15 ± 4.45
Respiratory Assistance Time (hours)	16.12 ± 42.16

As shown in Table 3, PPM was found to be severe in 54 patients (11.0%), moderate in 218 patients (44.4%), and absent in 219 patients (44.6%).

**Table 3:** PPM distribution based on severity PPM: Patient-prosthesis mismatch; None: Without PPM

PPM Degree	Number of Patients	Incidence Rate (%)
Severe	54	11.0%
Moderate	218	44.4%
None	219	44.6%
Total	491	100.0%

The overall mortality was 1.6%, with the highest rate observed in the patients with severe PPM (3.7%). Mortality rates based on PPM severity are shown in Table 4. The mortality rate was highest in the severe PPM group (3.7%, 2/54 patients), compared to 1.8% in the moderate (4/218) and 0.9% in the no PPM group (2/189) (p = 0.048).

**Table 4:** Mortality rate on PPM groups PPM: Patient-prosthesis mismatch; None: Without PPM

PPM Degree	Number of Patients	Deaths (Number)	Mortality Rate (%)
Severe	54	2	3.7%
Moderate	218	4	1.8%
None	219	2	0.9%
Total	491	8	1.6%

Results from the logistic regression analysis (including PPM and several established risk factors for early postoperative mortality) performed to investigate the impact of PPM on in-hospital mortality are summarized in Table 5. The regression model had good calibration (Hosmer-Lemeshow p = 0.41) and acceptable discrimination (c-statistic = 0.76). Severe PPM was identified as a significant predictor of early mortality (p = 0.050; OR, 15.62; 95% CI, 9.004-21.10), indicating that patients with severe PPM were 15.6 times more likely to die compared with those without PPM.

**Table 5:** Logistic regression for risk factors affecting mortality in isolated AVR EF: Ejection fraction; XC: Aortic cross-clamp; PPM: Patient-prosthesis mismatch; OR: Odds ratio; CI: Confidence interval; AVR: Aortic valve replacement p-value < 0.005; statistically significant

Variable	OR	95% CI (Lower-Upper)	p-value
EF	1.031	0.95-1.13	0.432
Gender (Male)	1.276	0.19-8.39	0.800
XC Time	1.030	0.97-1.09	0.331
PPM Severe	15.62	9.004-21.10	0.050
PPM moderate	0.201	0.01 – 2.86	0.237

## Discussion

Our study demonstrated a significant association between severe PPM and increased short-term mortality following isolated AVR. Specifically, patients with severe PPM exhibited a mortality rate of 3.7%, markedly higher than the 0.9% observed in patients without PPM. The significant OR of 15.62 in this study indicates a strong association between PPM severity and adverse outcomes. Although the absolute number of deaths was small (n=8), the effect size suggests a clinically meaningful association, warranting careful preoperative assessment.

The impact of severe PPM on early mortality is significant as the left ventricle (LV) is fragile, especially during the early postoperative period, and highly sensitive to even minor hemodynamic changes caused by the presence of severe PMM. Interestingly, although patients with moderate PPM had a higher absolute number of deaths (four cases), their mortality rate was lower than that of patients with severe PPM. This suggests that moderate PPM, through common, may not be as fatal as severe PPM.

Our findings are consistent with existing literature recognizing PPM as a critical determinant of post-AVR survival, further underscoring the significant impact of PPM on postoperative outcomes. A comprehensive meta-analysis by Michel de Oliveira et al., which encompassed 108,182 patients, demonstrated that PPM significantly increased perioperative, early, mid-term, and long-term mortality rates [3]. It also reported an overall PPM incidence of 53.7%, with severe PPM correlating with a higher risk of mortality compared with moderate PPM [3]. Similarly, Manacio et al. found a higher early mortality rate in the severe PPM group (3.7%) than the moderate PPM group (2.3%), though the difference was not statistically significant (p = 0.2) [9]. Rao et al., in a study involving 2,154 patients treated with AVR, reported that 30-day mortality was significantly higher in patients with evidence of PPM than in patients without PPM (7.9% vs 4.6%, p  <  0.05) [10]. These results reinforce our findings, highlighting the detrimental effect of severe PPM on patient survival.

Similarly, a 2018 study of 1,168 patients from the Valve-in-Valve International Data registry found that patients with severe PPM had higher 30-day and 1-year mortality rates compared with those without severe PPM (10.3% vs 4.3%, p = 0.01, and 19.3% vs 10.9%, p = 0.03, respectively) [11].

A meta-analysis of 58 studies examining not only perioperative but also long-term mortality among patients treated with isolated AVR for PPM (including 39,568 surgical AVR and 813 transcatheter AVR procedures) found that severe PPM was associated with an increased risk of both perioperative and overall mortality, while moderate PPM increased the risk of perioperative mortality without impacting overall mortality [12]. In contrast, a more recent study by Elhmahdy et al. found that the presence of PPM (either severe or moderate) was associated with a significant increase in 30-day mortality and had an independent association with all-cause mortality at a median follow-up of four years, leading to more than a twofold reduction in survival compared with no PPM [13].

The relationship between PPM and LV functional status is another significant argument addressed by Ruel et al. [14]. They demonstrated that PPM (defined as an EOA-i ≤ 0.85 cm²/m²) negatively impacted survival (p = 0.03), increased heart failure risk (p = 0.009), and impaired LV mass regression in patients with reduced LVEF (EF < 50%) versus those with normal LV function.

Additionally, patients with PPM and a reduced EF had a twofold higher risk of late mortality, a five-fold higher risk of heart failure, and no reduction in heart muscle mass compared with those with a reduced EF but no PPM. In the same context, a multifactorial analysis identified PPM as an independent risk factor for early mortality (p = 0.003), with its impact closely linked to both the severity of PPM and the degree of LV dysfunction [5].

Conversely, some studies have presented conflicting findings regarding the impact of PPM on early and late mortality, suggesting that its influence on survival may not be as detrimental as indicated by previous research. One such study, conducted by Tang et al., reported that although severe PPM was present in 5.3% of 42,174 patients who underwent transcatheter AVR for de novo stenosis and in 27.0% of 5,446 patients who received a transcatheter valve-in-surgical AV procedure, it was not significantly associated with one-year mortality or valve-related readmissions in either group [15]. These discrepancies may be attributable to differences in prosthetic valve design between transcatheter and surgically implanted valves. However, this remains speculative, as there is currently no definitive evidence establishing the superiority of one approach over the other in terms of PPM-related mortality.

Efforts to prevent PPM should include meticulous preoperative planning using CT-based annulus sizing and accurate prosthesis sizing. Alternative strategies to avoid patient-prosthesis mismatch (PPM) include: a) implanting a different type of prosthesis with a larger EOA-i (e.g., stentless bioprostheses, newer-generation mechanical valves, or aortic homografts); b) performing aortic root enlargement to allow implantation of a larger-sized prosthesis of the same type in borderline cases; and c) accepting PPM when necessary, after carefully weighing other clinical factors.

The choice among these alternatives must take into account the specific advantages and disadvantages of each approach. For example, in patients with reduced EF, aortic root enlargement or homograft implantation carries a high operative risk due to the technical complexity of these procedures. In such cases, if the initially planned prosthesis is a stented bioprosthesis, it may be preferable to switch to a mechanical valve or a stentless bioprosthesis, which generally offer better hemodynamic performance. However, stentless bioprostheses are currently not available in our country, limiting their use. If mechanical valve implantation is contraindicated, for instance, in patients with high bleeding risk, or if switching to a mechanical valve does not eliminate the PPM, then we accept the mismatch and manage its potential long-term effects accordingly. In our center, routine preoperative CT assessment and enlargement procedures remain underutilized and should be re-evaluated.

One limitation of this study is its retrospective, single-center design, which may restrict the generalization of the results. Additionally, the relatively small sample size may have limited the statistical power to detect subtle differences in outcomes. Also, the use of manufacturer-reported EOA values instead of in vivo echocardiographic measurements may limit clinical precision but aligns with common practices in large registries and prior literature. Importantly, patients with moderate PPM also comprised a substantial proportion of the cohort, highlighting the need for tailored strategies even in non-severe cases. The study also did not assess long-term survival or functional status beyond the 30-day postoperative period, nor did it account for factors such as left ventricular mass regression, which could influence the overall impact of PPM.

## Conclusions

PPM remains a common and relevant issue in AVR. This study found a strong link between severe PPM and higher early mortality, particularly in patients with already weakened LV function. While causality cannot be established, the findings underscore the importance of preoperative planning to minimize mismatch. Future studies should investigate long-term outcomes and evaluate the role of imaging and surgical strategies in PPM prevention. The findings support a more tailored approach to AVR, with the goal of optimizing both the technical and clinical results of the procedure.
